# 4-Acetyl­piperazinium picrate

**DOI:** 10.1107/S1600536814011726

**Published:** 2014-05-31

**Authors:** Channappa N. Kavitha, Manpreet Kaur, Jerry P. Jasinski, Hemmige S. Yathirajan

**Affiliations:** a Department of Studies in Chemistry, University of Mysore, Manasagangotri, Mysore 570 006, India; b Department of Chemistry, Keene State College, 229 Main Street, Keene, NH 03435-2001, USA

## Abstract

In the title salt, C_6_H_13_N_2_O^+^·C_6_H_2_N_3_O_7_^−^ (systematic name: 4-acetyl­piperazin-1-ium 2,4,6-tri­nitro­phenolate), the piperazin-1-ium ring has a slightly distorted chair conformation. In the picrate anion, the mean planes of the two *o*-NO_2_ and *p*-NO_2_ groups are twisted with respect to the benzene ring by 15.0 (2), 68.9 (4) and 4.4 (3)°, respectively. In the crystal, N—H⋯O hydrogen bonds are observed, linking the ions into an infinite chain along [010]. In addition, weak cation–anion C—H⋯O inter­molecular inter­actions and a weak π–π stacking inter­action between the benzene rings of the anions, with an inter-centroid distance of 3.771 (8) Å, help to stabilize the crystal packing, giving an overall sheet structure lying parallel to (100). Disorder was modelled for one of the O atoms in one of the *o*-NO_2_ groups over two sites with an occupancy ratio of 0.57 (6):0.43 (6).

## Related literature   

Piperazines and substituted piperazines are important pharmacophores that can be found in many biologically active compounds across a number of different therapeutic areas, see: Berkheij (2005 ▶); Choudhary *et al.* (2006 ▶); Kharb *et al.* (2012 ▶); Upadhayaya *et al.* (2004 ▶). For picric acid salts, see: Hundal *et al.* (1997 ▶); Szumna *et al.* (2000 ▶); Colquhoun *et al.* (1986 ▶). For related structures, see: Kavitha *et al.* (2013 ▶, 2014 ▶); Loughlin *et al.* (2003 ▶); Wang & Jia (2008 ▶); Song *et al.* (2012 ▶). For puckering parameters, see Cremer & Pople (1975 ▶). For standard bond lengths, see: Allen *et al.* (1987 ▶).
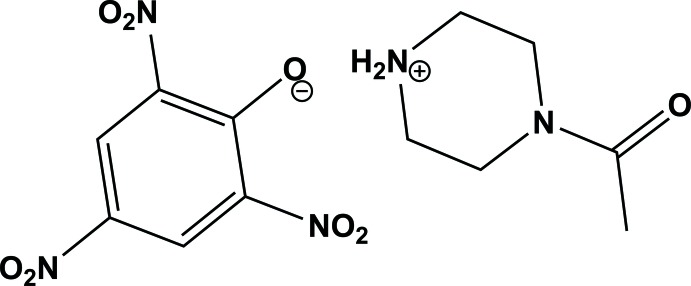


## Experimental   

### 

#### Crystal data  

C_6_H_13_N_2_O^+^·C_6_H_2_N_3_O_7_^−^
*M* *_r_* = 357.29
Monoclinic, 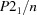

*a* = 6.6843 (7) Å
*b* = 11.5971 (12) Å
*c* = 20.131 (2) Å
β = 90.000 (4)°
*V* = 1560.5 (3) Å^3^
*Z* = 4
Cu *K*α radiation
μ = 1.12 mm^−1^
*T* = 173 K
0.32 × 0.28 × 0.06 mm

#### Data collection  

Agilent Eos Gemini diffractometer
Absorption correction: multi-scan (*CrysAlis PRO*; Agilent, 2014 ▶) *T*_min_ = 0.631, *T*_max_ = 1.000
9739 measured reflections
2993 independent reflections
2690 reflections with *I* > 2σ(*I*)
*R*_int_ = 0.036

#### Refinement  

*R*[*F* ^2^ > 2σ(*F* ^2^)] = 0.045
*wR*(*F* ^2^) = 0.126
*S* = 1.10
2993 reflections
238 parameters
H-atom parameters constrained
Δρ_max_ = 0.27 e Å^−3^
Δρ_min_ = −0.20 e Å^−3^

### 

Data collection: *CrysAlis PRO* (Agilent, 2014 ▶); cell refinement: *CrysAlis PRO*; data reduction: *CrysAlis PRO*; program(s) used to solve structure: *SUPERFLIP* (Palatinus & Chapuis, 2007 ▶); program(s) used to refine structure: *SHELXL97* (Sheldrick, 2008 ▶); molecular graphics: *OLEX2* (Dolomanov *et al.*, 2009 ▶); software used to prepare material for publication: *OLEX2*.

## Supplementary Material

Crystal structure: contains datablock(s) I. DOI: 10.1107/S1600536814011726/zs2300sup1.cif

Structure factors: contains datablock(s) I. DOI: 10.1107/S1600536814011726/zs2300Isup2.hkl

Supporting information file. DOI: 10.1107/S1600536814011726/zs2300Isup3.cml

CCDC reference: 1004370

Additional supporting information:  crystallographic information; 3D view; checkCIF report

## Figures and Tables

**Table 1:** Hydrogen-bond geometry (Å, °)

*D*—H⋯*A*	*D*—H	H⋯*A*	*D*⋯*A*	*D*—H⋯*A*
N2*A*—H2*AA*⋯O1*A* ^i^	0.97	1.78	2.7057 (19)	159
N2*A*—H2*AB*⋯O1*B* ^ii^	0.97	1.82	2.7401 (19)	157
C3*A*—H3*AA*⋯O5*B* ^i^	0.97	2.46	3.333 (2)	150
C3*A*—H3*AB*⋯O3*B* ^iii^	0.97	2.55	3.469 (3)	158
C5*A*—H5*AA*⋯O7*B* ^iv^	0.97	2.57	3.365 (2)	139
C5*B*—H5*B*⋯O1*A*	0.93	2.47	3.307 (2)	149

Symmetry codes: (i) 
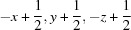
; (ii) 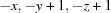; (iii) 
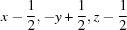
; (iv) 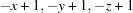.

**Fig. 1.**
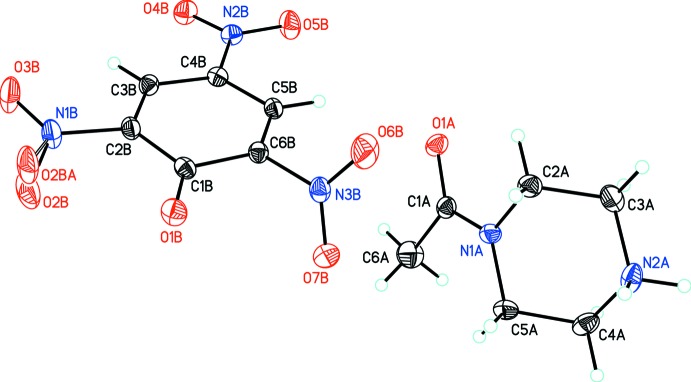
ORTEP drawing of the title compound showing the labeling scheme with 30% probability displacement ellipsoids.

**Fig. 2.**
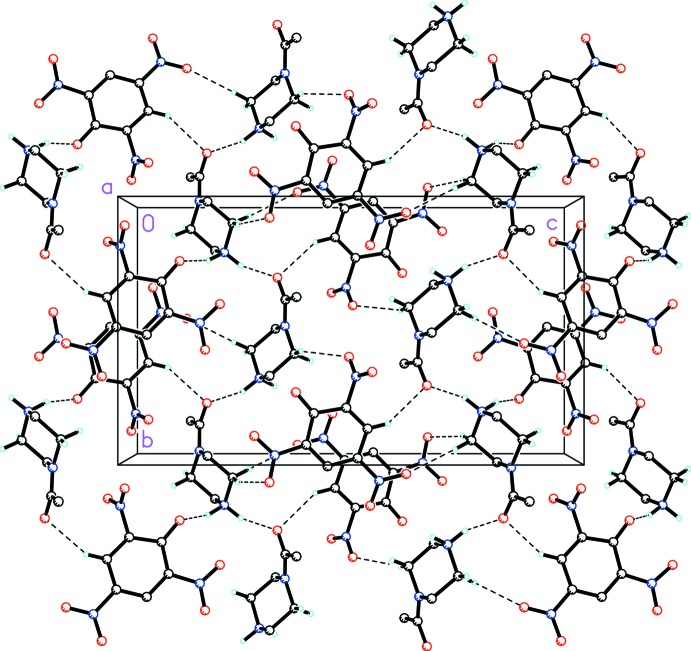
Molecular packing viewed along the a axis. Dashed lines indicate N—H···O intermolecular hydrogen bonds forming infinite one-dimensional chains along [0 1 0] and further supported by weak C—H···O intermolecular interactions. H atoms not involved in hydrogen bonding have been removed for clarity. The disordered component of the C2 o-NO2 group is also omitted.

## supplementary crystallographic information

### 1. Comment

Piperazines and substituted piperazines are important pharmacophores that can be found in many biologically active compounds across a number of different therapeutic areas (Berkheij, 2005) such as antifungal (Upadhayaya *et al.*, 2004), anti-bacterial, anti-malarial and anti-psychotic agents (Choudhary *et al.*, 2006). A valuable insight into recent advances on antimicrobial activity of piperazine derivatives has been reported (Kharb *et al.*, 2012). Also picric acid forms salts which exhibit electrostatic forces, multiple hydrogen bonds (Hundal *et al.*, 1997; Szumna *et al.*, 2000) and π–π stacking interactions (Colquhoun *et al.*, 1986), which improve the quality of the crystalline materials. The supra-molecular structure of molecular adducts of picric acid and piperazine have been reported (Wang & Jia, 2008). The crystal structures of some related compounds, viz., 1-[4-(4-hydroxyphenyl)piperazin-1-yl]ethanone (Kavitha *et al.*, 2013), 3-(Z)-isobutylidene-1-acetylpiperazine-2,5-dione (Loughlin *et al.* , 2003), piperazine-1,4-diium picrate-piperazine (Wang & Jia, 2008), cinnarizinium picrate (Song *et al.*, 2012) and 1-piperonylpiperazinium picrate (Kavitha *et al.*, 2014) have been reported. In view of the importance of the title compound, C_6_H_13_N_2_O^+^. C_6_H_2_N_3_O_7_^-^, this paper reports its crystal structure.

The title salt crystallizes with one piperazinium cation (*A*) and a picrate anion (*B*) in the asymmetric unit (Fig. 1). In the cation, the piperazine ring is in a slightly distorted chair conformation (puckering parameters Q, θ, and φ = 0.569 (2)Å, 178.3 (5)° and 197 (9)°, respectively (Cremer & Pople, 1975). In the picrate anion, the mean planes of the two *o*-NO_2_ groups and the *p*-NO_2_ group are twisted with respect to the phenyl ring plane by 15.0 (2)°, 68.9 (4)° and 4.4 (3)°, respectively. Bond lengths are in normal ranges (Allen *et al.*, 1987). Intermolecular N—H···O hydrogen bonds are observed (Table 1) linking the anions with the cations and other anions forming an infinite one-dimensional chain along [010] (Fig. 2). In addition, weak cation-anion intermolecular C—H···O interactions and a weak π–π stacking interaction between the anionic phenyl rings [inter-centroid distance = 3.771 (8) Å] stabilize the crystal packing and generate a overall two-dimensional sheet structure lying parallel to (100). Disorder was modelled for the O2*B* oxygen atom in one of the *o*-NO_2_ groups over two sites with an occupancy ratio of 0.57 (6):0.43 (6).

### 2. Experimental

Picric acid (1.14 g, 0.005 mol) was dissolved in methanol and acetyl piperazine (0.63 ml, 0.005 mol) was added to it with stirring. A yellow precipitate was obtained instantaneously. The precipitate was recrystallized from ethanol by slow evaporation (m.p.: 443–448 K).

### 3. Refinement

All of the H atoms were placed in their calculated positions and then refined using the riding model with atom—H lengths of 0.93 Å(CH); 0.97 Å (CH_2_); 0.96 Å (CH_3_) or 0.97 Å (NH). Isotropic displacement parameters for these atoms were set to 1.2 (CH, CH_2_, NH) or 1.5 (CH_3_) times *U*_eq_ of the parent atom. The methyl group was refined as a rotating group. Disorder was modelled for O2B in one of the *o*-NO_2_ groups over two sites with an occupancy ratio of 0.57 (6):0.43 (6). The incorrect orthorhombic unit cell was transformed into the correct monoclinic *P*2_1_/*n* cell having β = 90.000 (4)°, which prompted the *checkCIF/PLATON* B-ALERT (SYMMS 02).

### Crystal data

**Table:**

C_6_H_13_N_2_O^+^·C_6_H_2_N_3_O_7_^−^	*D*_x_ = 1.521 Mg m^−^^3^
*M**_r_* = 357.29	Melting point = 443–448 K
Monoclinic, *P*2_1_/*n*	Cu *K*α radiation, λ = 1.54184 Å
*a* = 6.6843 (7) Å	Cell parameters from 4582 reflections
*b* = 11.5971 (12) Å	θ = 4.4–71.6°
*c* = 20.131 (2) Å	µ = 1.12 mm^−^^1^
β = 90.000 (4)°	*T* = 173 K
*V* = 1560.5 (3) Å^3^	Block, yellow
*Z* = 4	0.32 × 0.28 × 0.06 mm
*F*(000) = 744	

### Data collection

**Table:**

Agilent Eos Gemini diffractometer	2993 independent reflections
Radiation source: Enhance (Cu) X-ray Source	2690 reflections with *I* > 2σ(*I*)
Detector resolution: 16.0416 pixels mm^-1^	*R*_int_ = 0.036
ω scans	θ_max_ = 72.0°, θ_min_ = 4.4°
Absorption correction: multi-scan (*CrysAlis PRO*; Agilent, 2014)	*h* = −8→7
*T*_min_ = 0.631, *T*_max_ = 1.000	*k* = −14→11
9739 measured reflections	*l* = −24→24

### Refinement

**Table:**

Refinement on *F*^2^	Hydrogen site location: inferred from neighbouring sites
Least-squares matrix: full	H-atom parameters constrained
*R*[*F*^2^ > 2σ(*F*^2^)] = 0.045	*w* = 1/[σ^2^(*F*_o_^2^) + (0.0624*P*)^2^ + 0.6066*P*] where *P* = (*F*_o_^2^ + 2*F*_c_^2^)/3
*wR*(*F*^2^) = 0.126	(Δ/σ)_max_ < 0.001
*S* = 1.10	Δρ_max_ = 0.27 e Å^−^^3^
2993 reflections	Δρ_min_ = −0.20 e Å^−^^3^
238 parameters	Extinction correction: *SHELXL97* (Sheldrick, 2008), Fc^*^=kFc[1+0.001xFc^2^λ^3^/sin(2θ)]^-1/4^
0 restraints	Extinction coefficient: 0.0018 (3)
Primary atom site location: structure-invariant direct methods	

### Special details

**Table:**

Experimental. Absorption correction: CrysAlis PRO (Agilent, 2014), Version 1.171.37.31 (release 14-01-2014 CrysAlis171 .NET) (compiled Jan 14 2014,18:38:05) Empirical absorption correction using spherical harmonics, implemented in SCALE3 ABSPACK scaling algorithm.
Geometry. All esds (except the esd in the dihedral angle between two l.s. planes) are estimated using the full covariance matrix. The cell esds are taken into account individually in the estimation of esds in distances, angles and torsion angles; correlations between esds in cell parameters are only used when they are defined by crystal symmetry. An approximate (isotropic) treatment of cell esds is used for estimating esds involving l.s. planes.

### Fractional atomic coordinates and isotropic or equivalent isotropic displacement parameters (Å^2^)

**Table:**

	*x*	*y*	*z*	*U*_iso_*/*U*_eq_	Occ. (<1)
O1B	0.10430 (19)	0.26818 (11)	0.61927 (6)	0.0352 (3)	
O2B	0.175 (7)	0.1019 (8)	0.7106 (4)	0.076 (5)	0.57 (6)
O2BA	0.082 (4)	0.0947 (14)	0.7071 (7)	0.051 (4)	0.43 (6)
O3B	0.1631 (3)	−0.07329 (14)	0.67738 (7)	0.0541 (4)	
O4B	0.3371 (2)	−0.17212 (11)	0.45759 (7)	0.0395 (3)	
O5B	0.3065 (2)	−0.04833 (12)	0.37818 (6)	0.0434 (3)	
O6B	−0.0003 (2)	0.34623 (15)	0.45462 (9)	0.0573 (4)	
O7B	0.2665 (3)	0.39977 (12)	0.50421 (8)	0.0551 (4)	
N1B	0.1575 (2)	0.02945 (14)	0.66573 (7)	0.0378 (4)	
N2B	0.3023 (2)	−0.07436 (12)	0.43751 (7)	0.0291 (3)	
N3B	0.1445 (2)	0.32723 (12)	0.48878 (7)	0.0304 (3)	
C1B	0.1524 (2)	0.18809 (14)	0.58083 (8)	0.0255 (3)	
C2B	0.1827 (2)	0.06833 (15)	0.59752 (8)	0.0274 (4)	
C3B	0.2322 (2)	−0.01538 (14)	0.55123 (8)	0.0259 (3)	
H3B	0.2513	−0.0913	0.5646	0.031*	
C4B	0.2531 (2)	0.01431 (14)	0.48536 (8)	0.0247 (3)	
C5B	0.2228 (2)	0.12757 (14)	0.46350 (7)	0.0249 (3)	
H5B	0.2332	0.1469	0.4188	0.030*	
C6B	0.1776 (2)	0.20831 (13)	0.51029 (8)	0.0244 (3)	
O1A	0.31825 (19)	0.29011 (10)	0.33233 (6)	0.0341 (3)	
N1A	0.3133 (2)	0.48149 (12)	0.34934 (7)	0.0290 (3)	
N2A	0.1899 (2)	0.69006 (13)	0.28906 (7)	0.0359 (4)	
H2AA	0.1923	0.7422	0.2514	0.043*	
H2AB	0.1046	0.7237	0.3229	0.043*	
C1A	0.4034 (2)	0.37845 (14)	0.35166 (8)	0.0282 (4)	
C2A	0.1095 (3)	0.49259 (15)	0.32500 (9)	0.0328 (4)	
H2AC	0.0231	0.5196	0.3605	0.039*	
H2AD	0.0609	0.4180	0.3103	0.039*	
C3A	0.1047 (3)	0.57715 (17)	0.26784 (9)	0.0389 (4)	
H3AA	0.1816	0.5468	0.2309	0.047*	
H3AB	−0.0322	0.5878	0.2531	0.047*	
C4A	0.3952 (3)	0.67764 (15)	0.31576 (9)	0.0365 (4)	
H4AA	0.4425	0.7516	0.3318	0.044*	
H4AB	0.4845	0.6520	0.2808	0.044*	
C5A	0.3959 (3)	0.59116 (15)	0.37192 (9)	0.0361 (4)	
H5AA	0.5318	0.5797	0.3875	0.043*	
H5AB	0.3168	0.6204	0.4086	0.043*	
C6A	0.6101 (3)	0.37046 (19)	0.37903 (12)	0.0475 (5)	
H6AA	0.6105	0.3967	0.4242	0.071*	
H6AB	0.6984	0.4177	0.3531	0.071*	
H6AC	0.6545	0.2918	0.3774	0.071*	

### Atomic displacement parameters (Å^2^)

**Table:**

	*U*^11^	*U*^22^	*U*^33^	*U*^12^	*U*^13^	*U*^23^
O1B	0.0435 (7)	0.0337 (7)	0.0286 (6)	0.0049 (5)	0.0062 (5)	−0.0059 (5)
O2B	0.143 (15)	0.062 (3)	0.0223 (16)	−0.009 (4)	−0.002 (4)	−0.0016 (14)
O2BA	0.089 (9)	0.044 (4)	0.021 (3)	0.014 (3)	0.014 (3)	0.002 (2)
O3B	0.0824 (11)	0.0453 (9)	0.0347 (7)	0.0073 (7)	0.0086 (7)	0.0161 (6)
O4B	0.0513 (8)	0.0266 (6)	0.0406 (7)	0.0050 (5)	0.0031 (6)	−0.0035 (5)
O5B	0.0643 (9)	0.0419 (7)	0.0241 (6)	0.0098 (6)	0.0067 (6)	−0.0033 (5)
O6B	0.0440 (8)	0.0534 (9)	0.0744 (11)	0.0097 (7)	−0.0153 (7)	0.0236 (8)
O7B	0.0842 (11)	0.0311 (7)	0.0501 (9)	−0.0127 (7)	−0.0205 (8)	0.0036 (6)
N1B	0.0489 (9)	0.0413 (9)	0.0232 (7)	0.0041 (7)	0.0007 (6)	0.0058 (6)
N2B	0.0293 (7)	0.0285 (7)	0.0295 (7)	0.0008 (5)	0.0022 (5)	−0.0038 (6)
N3B	0.0381 (8)	0.0290 (7)	0.0240 (7)	0.0052 (6)	0.0020 (6)	0.0007 (5)
C1B	0.0222 (7)	0.0325 (8)	0.0218 (7)	0.0007 (6)	−0.0001 (5)	−0.0021 (6)
C2B	0.0287 (8)	0.0327 (9)	0.0206 (8)	−0.0008 (6)	0.0002 (6)	0.0029 (6)
C3B	0.0238 (7)	0.0260 (8)	0.0280 (8)	0.0007 (6)	−0.0008 (6)	0.0035 (6)
C4B	0.0213 (7)	0.0276 (8)	0.0252 (8)	0.0005 (6)	0.0013 (6)	−0.0022 (6)
C5B	0.0248 (7)	0.0295 (8)	0.0206 (7)	0.0000 (6)	−0.0001 (5)	0.0014 (6)
C6B	0.0229 (7)	0.0257 (8)	0.0244 (8)	0.0014 (6)	−0.0007 (5)	0.0016 (6)
O1A	0.0473 (7)	0.0235 (6)	0.0315 (6)	−0.0022 (5)	0.0039 (5)	−0.0024 (5)
N1A	0.0326 (7)	0.0235 (7)	0.0308 (7)	−0.0001 (5)	−0.0057 (6)	−0.0032 (5)
N2A	0.0523 (9)	0.0291 (7)	0.0263 (7)	0.0109 (6)	0.0108 (6)	0.0039 (6)
C1A	0.0353 (9)	0.0263 (8)	0.0229 (7)	0.0008 (6)	0.0029 (6)	0.0005 (6)
C2A	0.0310 (8)	0.0300 (8)	0.0375 (9)	0.0006 (6)	−0.0050 (7)	−0.0014 (7)
C3A	0.0435 (10)	0.0408 (10)	0.0323 (9)	0.0081 (8)	−0.0078 (7)	−0.0026 (7)
C4A	0.0464 (10)	0.0240 (8)	0.0391 (10)	−0.0024 (7)	0.0085 (8)	−0.0068 (7)
C5A	0.0462 (10)	0.0276 (9)	0.0345 (9)	−0.0033 (7)	−0.0084 (7)	−0.0076 (7)
C6A	0.0388 (11)	0.0455 (11)	0.0582 (13)	0.0096 (8)	−0.0082 (9)	−0.0002 (9)

### Geometric parameters (Å, º)

**Table:**

O1B—C1B	1.251 (2)	N1A—C2A	1.453 (2)
O2B—N1B	1.239 (7)	N1A—C5A	1.459 (2)
O2BA—N1B	1.231 (10)	N2A—H2AA	0.9700
O3B—N1B	1.215 (2)	N2A—H2AB	0.9700
O4B—N2B	1.2260 (19)	N2A—C3A	1.490 (2)
O5B—N2B	1.232 (2)	N2A—C4A	1.481 (3)
O6B—N3B	1.208 (2)	C1A—C6A	1.490 (3)
O7B—N3B	1.212 (2)	C2A—H2AC	0.9700
N1B—C2B	1.455 (2)	C2A—H2AD	0.9700
N2B—C4B	1.447 (2)	C2A—C3A	1.512 (3)
N3B—C6B	1.462 (2)	C3A—H3AA	0.9700
C1B—C2B	1.443 (2)	C3A—H3AB	0.9700
C1B—C6B	1.449 (2)	C4A—H4AA	0.9700
C2B—C3B	1.386 (2)	C4A—H4AB	0.9700
C3B—H3B	0.9300	C4A—C5A	1.511 (3)
C3B—C4B	1.377 (2)	C5A—H5AA	0.9700
C4B—C5B	1.400 (2)	C5A—H5AB	0.9700
C5B—H5B	0.9300	C6A—H6AA	0.9600
C5B—C6B	1.362 (2)	C6A—H6AB	0.9600
O1A—C1A	1.235 (2)	C6A—H6AC	0.9600
N1A—C1A	1.339 (2)		
			
O2B—N1B—C2B	117.9 (5)	C4A—N2A—H2AB	109.2
O2BA—N1B—C2B	119.6 (4)	C4A—N2A—C3A	111.88 (13)
O3B—N1B—O2B	121.4 (4)	O1A—C1A—N1A	121.49 (15)
O3B—N1B—O2BA	119.0 (6)	O1A—C1A—C6A	119.48 (16)
O3B—N1B—C2B	118.85 (15)	N1A—C1A—C6A	119.03 (16)
O4B—N2B—O5B	122.81 (14)	N1A—C2A—H2AC	109.8
O4B—N2B—C4B	118.74 (14)	N1A—C2A—H2AD	109.8
O5B—N2B—C4B	118.45 (14)	N1A—C2A—C3A	109.51 (15)
O6B—N3B—O7B	123.95 (16)	H2AC—C2A—H2AD	108.2
O6B—N3B—C6B	117.50 (15)	C3A—C2A—H2AC	109.8
O7B—N3B—C6B	118.50 (14)	C3A—C2A—H2AD	109.8
O1B—C1B—C2B	127.34 (15)	N2A—C3A—C2A	110.09 (15)
O1B—C1B—C6B	121.06 (15)	N2A—C3A—H3AA	109.6
C2B—C1B—C6B	111.57 (14)	N2A—C3A—H3AB	109.6
C1B—C2B—N1B	120.12 (14)	C2A—C3A—H3AA	109.6
C3B—C2B—N1B	116.43 (15)	C2A—C3A—H3AB	109.6
C3B—C2B—C1B	123.44 (14)	H3AA—C3A—H3AB	108.2
C2B—C3B—H3B	120.1	N2A—C4A—H4AA	109.7
C4B—C3B—C2B	119.78 (15)	N2A—C4A—H4AB	109.7
C4B—C3B—H3B	120.1	N2A—C4A—C5A	109.82 (15)
C3B—C4B—N2B	119.11 (14)	H4AA—C4A—H4AB	108.2
C3B—C4B—C5B	121.50 (14)	C5A—C4A—H4AA	109.7
C5B—C4B—N2B	119.36 (14)	C5A—C4A—H4AB	109.7
C4B—C5B—H5B	121.3	N1A—C5A—C4A	110.13 (14)
C6B—C5B—C4B	117.37 (14)	N1A—C5A—H5AA	109.6
C6B—C5B—H5B	121.3	N1A—C5A—H5AB	109.6
C1B—C6B—N3B	115.17 (13)	C4A—C5A—H5AA	109.6
C5B—C6B—N3B	118.50 (14)	C4A—C5A—H5AB	109.6
C5B—C6B—C1B	126.31 (15)	H5AA—C5A—H5AB	108.1
C1A—N1A—C2A	120.82 (14)	C1A—C6A—H6AA	109.5
C1A—N1A—C5A	126.65 (14)	C1A—C6A—H6AB	109.5
C2A—N1A—C5A	112.49 (14)	C1A—C6A—H6AC	109.5
H2AA—N2A—H2AB	107.9	H6AA—C6A—H6AB	109.5
C3A—N2A—H2AA	109.2	H6AA—C6A—H6AC	109.5
C3A—N2A—H2AB	109.2	H6AB—C6A—H6AC	109.5
C4A—N2A—H2AA	109.2		
			
O1B—C1B—C2B—N1B	0.0 (3)	C2B—C1B—C6B—N3B	178.55 (13)
O1B—C1B—C2B—C3B	178.68 (15)	C2B—C1B—C6B—C5B	0.4 (2)
O1B—C1B—C6B—N3B	0.5 (2)	C2B—C3B—C4B—N2B	−179.20 (13)
O1B—C1B—C6B—C5B	−177.66 (15)	C2B—C3B—C4B—C5B	−0.9 (2)
O2B—N1B—C2B—C1B	−23 (2)	C3B—C4B—C5B—C6B	1.9 (2)
O2B—N1B—C2B—C3B	158 (2)	C4B—C5B—C6B—N3B	−179.80 (13)
O2BA—N1B—C2B—C1B	10.6 (19)	C4B—C5B—C6B—C1B	−1.7 (2)
O2BA—N1B—C2B—C3B	−168.2 (18)	C6B—C1B—C2B—N1B	−177.95 (14)
O3B—N1B—C2B—C1B	172.35 (17)	C6B—C1B—C2B—C3B	0.8 (2)
O3B—N1B—C2B—C3B	−6.5 (2)	N1A—C2A—C3A—N2A	−56.25 (19)
O4B—N2B—C4B—C3B	−4.8 (2)	N2A—C4A—C5A—N1A	55.87 (19)
O4B—N2B—C4B—C5B	176.84 (14)	C1A—N1A—C2A—C3A	−123.25 (17)
O5B—N2B—C4B—C3B	174.87 (15)	C1A—N1A—C5A—C4A	123.37 (18)
O5B—N2B—C4B—C5B	−3.5 (2)	C2A—N1A—C1A—O1A	1.2 (2)
O6B—N3B—C6B—C1B	−111.79 (18)	C2A—N1A—C1A—C6A	−178.04 (17)
O6B—N3B—C6B—C5B	66.5 (2)	C2A—N1A—C5A—C4A	−59.09 (19)
O7B—N3B—C6B—C1B	70.6 (2)	C3A—N2A—C4A—C5A	−55.55 (18)
O7B—N3B—C6B—C5B	−111.05 (18)	C4A—N2A—C3A—C2A	56.01 (19)
N1B—C2B—C3B—C4B	178.21 (14)	C5A—N1A—C1A—O1A	178.54 (16)
N2B—C4B—C5B—C6B	−179.75 (13)	C5A—N1A—C1A—C6A	−0.7 (3)
C1B—C2B—C3B—C4B	−0.5 (2)	C5A—N1A—C2A—C3A	59.05 (19)

### Hydrogen-bond geometry (Å, º)

**Table:**

*D*—H···*A*	*D*—H	H···*A*	*D*···*A*	*D*—H···*A*
N2*A*—H2*AA*···O1*A*^i^	0.97	1.78	2.7057 (19)	159
N2*A*—H2*AB*···O1*B*^ii^	0.97	1.82	2.7401 (19)	157
C3*A*—H3*AA*···O5*B*^i^	0.97	2.46	3.333 (2)	150
C3*A*—H3*AB*···O3*B*^iii^	0.97	2.55	3.469 (3)	158
C5*A*—H5*AA*···O7*B*^iv^	0.97	2.57	3.365 (2)	139
C5*B*—H5*B*···O1*A*	0.93	2.47	3.307 (2)	149
C5*A*—H5*AB*···O4*B*^v^	0.97	2.60	3.266 (2)	126

Symmetry codes: (i) −*x*+1/2, *y*+1/2, −*z*+1/2; (ii) −*x*, −*y*+1, −*z*+1; (iii) *x*−1/2, −*y*+1/2, *z*−1/2; (iv) −*x*+1, −*y*+1, −*z*+1; (v) *x*, *y*+1, *z*.
